# Editorial: Progress in understanding and treating distress, sleep, and personality in heart and lung disease

**DOI:** 10.3389/fpsyg.2023.1323677

**Published:** 2023-11-07

**Authors:** John Munkhaugen, Costas Papageorgiou, Roger Hagen, Sverre Urnes Johnson, Gunnar Einvik, Toril Dammen

**Affiliations:** ^1^Department of Medicine, Drammen Hospital, Vestre Viken Trust, Drammen, Norway; ^2^Department of Behavioural Medicine, Institute of Basic Medical Sciences, University of Oslo, Oslo, Norway; ^3^Department of Psychology, University of Oslo, Oslo, Norway; ^4^Department of Psychology, Norwegian University of Science and Technology, Trondheim, Norway; ^5^Modum Bad Psychiatric Center, Vikersund, Norway; ^6^Faculty of Medicine, Institute of Clinical Medicine, University of Oslo, Oslo, Norway; ^7^Pulmonary Department, Akershus University Hospital, Lørenskog, Norway; ^8^Division of Mental Health and Addiction, Department of Research and Innovation, Oslo University Hospital, Oslo, Norway

**Keywords:** anxiety, depression, type D personality, sleep, metacognition, coronary heart disease, lung disease, treatment

Heart and lung diseases are leading causes of morbidity and mortality worldwide (Halpin et al., 2019; Smaardijk et al., 2019). There is a growing body of evidence pointing to the presence of prevalent comorbid conditions such as anxiety, depression, sleep disturbances, and type D personality. These factors significantly influence treatment adherence, quality of life, and prognosis in these patients. This Research Topic was initiated to present novel research that advance our understanding on the role of these factors in patients with cardiovascular and pulmonary diseases, with the ultimate goal of improving outcomes.

Health-related quality of life (HRQoL) is an important outcome in patients with cardiovascular and pulmonary disease. In a comprehensive cross-sectional multicenter study from Norway, Frøjd et al. identified the determinants of the mental and physical component scores of HRQoL in 1,042 coronary heart disease (CHD) outpatients. Type D personality was present in 18% of this sample, more than one third had significant symptoms of depression or anxiety, whereas 45% met self-reported diagnostic criteria for insomnia. Type D personality, depressive symptoms, and insomnia remained as factors associated with lower mental component scores of HRQoL whereas physical inactivity was the only potentially modifiable factor associated with lower physical component scores. The results suggest that physical activity and management of psychological distress and insomnia may improve HRQoL in CHD outpatients.

In confirmatory factor and regression analyses within a structural equation modeling framework of the same CHD population, Tunheim et al. showed that depression, anxiety, and the Type-D personality characteristics of negative affectivity and social inhibition all had acceptable factor structure, and an overlap emerged between the constructs of depression and negative affectivity. Further, worry was most strongly associated with anxiety whereas rumination was most strongly associated with depression and negative affectivity. The results indicate conceptual similarities across the measures of depression and negative affectivity and suggest that intervention efforts could benefit from targeting worry and/or rumination in the treatment of CHD outpatients presenting with symptoms of psychological distress.

In a longitudinal analysis conducted in the same CHD population, Torgersen et al. found higher scores on negative affectivity, assessed longer time after a CHD event, to be associated with increased risk of recurrent cardiovascular events following a mean 4.2 years follow-up, even after adjustment for age, cardiovascular risk factors and comorbidity, but not after controlling for anxiety and depression. Daily smoking and non-adherence to lipid lowering treatment were more prevalent in patients with high negative affectivity/type D personality than in those without. These data suggested that screening for these characteristics even a longer time after a CHD event may aid in identifying a high-risk CHD subgroup in need of individualized treatment and close follow-up care.

Despite anxiety and depression being common in patients with CHD, most psychological treatments have shown limited effectiveness in this patient group. In an open study, Dammen, Tunheim, Munkhaugen, Papageorgiou evaluated the acceptability and feasibility of the Attention Training Technique (ATT; Wells, 1990), a key component of metacognitive therapy, delivered weekly in a group format to CHD patients. The attendance rate was high and clinically significant improvements in symptoms of anxiety, depression, worry, rumination, and insomnia were reported from pre-treatment to 6-months follow-up. The authors concluded that the next step is to test the effectiveness of group ATT in an adequately powered study. Accordingly, Dammen, Tunheim, Munkhaugen, Klungsøyr et al. described the rationale and methodology of a larger multicenter trial in CHD patients that will be randomized into ATT or wait-list control arms. Changes in anxiety and depression scores between treatment arms will be the primary outcome whereas secondary outcomes will include changes in psychiatric disorders, rumination, worry, type D-personality, metacognitions, insomnia, quality of life, and subclinical inflammation, which is a risk marker for CHD.

In a qualitative study, Getz et al. explored the facilitators and barriers to smoking cessation in 10 CHD patients who participated in a cessation intervention comprising an in-hospital motivational interview, access to pharmacological treatment free of charge, and continuous follow-up after discharge. Five patients stopped smoking and five had relapsed/continued. The main barriers identified included: the upsides of smoking, difficult life situations, addiction to smoking, smoking in social circles, perceived lack of support, and understanding from healthcare professionals. Facilitators included intrinsic motivation, concerns about the health condition, financial implications, specific behavioral strategies, positive influence from the social environment, and helpful components of the cessation intervention. These facilitators and barriers may be targets for future cessation interventions.

Psychological distress is also highly prevalent among patients with chronic obstructive pulmonary disease (COPD; e.g., Marsh and Guck, 2016). The Metacognitions Questionnaire-30 (MCQ-30) assesses metacognitive beliefs and processes that are central components of the metacognitive model of emotional disorders (Wells and Matthews, 1994). The psychometric properties of the MCQ-30 in COPD patients have been unknown. Therefore, Dammen, Papageorgiou et al. administered the MCQ-30 to 203 COPD patients referred to a rehabilitation unit in respiratory medicine. Confirmatory factor analyses revealed that the factors were mostly moderately correlated and the exploratory analysis identified three of the five factors originally described in the five-factor model of the MCQ-30. Thus, the factor structure of the MCQ-30 in COPD patients appeared to differ from the original instrument, but the authors cautioned against any firm conclusions before further psychometric studies are conducted.

Contrary to insomnia and other sleep disorders, it remains uncertain whether sleep complaints are associated with mortality risk. Wang et al. aimed to investigate this relationship by linking data from 28,000 US adults participating in National Health and Nutrition Examination Survey to the national death registry. During >9 years follow-up, sleep complaints measured by screening questions, were associated with a 17% increased risk of all-cause mortality in the total population and a 24% risk of cardiovascular mortality in the subgroup with cardiovascular disease or cancer. Interestingly, a sub-analysis revealed that sleep complaints mainly increased mortality risk in those with a sleep duration <8 hours/day. Thus, monitoring and managing sleep complaints in addition to sleep disorders might be justified in a public health perspective.

In summary, this Research Topic offers the readers: (a) novel insights into the role of psychological distress, sleep, personality in patients with and without cardiovascular disease; (b) evaluation of assessment tools to measure distress, personality, and sleep, and (c) feasibility and preliminary data of the ATT applied in a group format in CHD patients. In a longer-term perspective, this evidence may improve our ability to screen or assess these conditions in the routine clinical setting and, importantly, develop and implement empirically grounded effective interventions that improve outcomes for the increasing patient-population with heart and lung diseases.

## Author contributions

JM: Writing – original draft, Writing – review & editing. CP: Writing – review & editing. RH: Writing – review & editing. SJ: Writing – review & editing. GE: Writing – review & editing. TD: Writing – review & editing.
